# The Rarest Subtype of Plasma Cell Dyscrasia: IgE Multiple Myeloma

**DOI:** 10.7759/cureus.91401

**Published:** 2025-09-01

**Authors:** Nora El Maachi, Soukaina Haidouri, Naoufal Benlachgar, Belkhayat Aziza, Zoubida Tazi Mezalek

**Affiliations:** 1 Hematology, Mohammed V Military Hospital, Rabat, MAR; 2 Hematology, Centre Hospitalo-Universitaire Avicenne, Rabat, MAR; 3 Laboratory Medicine, BIOLAB Laboratory, Rabat, MAR; 4 Internal Medicine and Hematology, Ibn Sina Hospital, Mohammed V University, Rabat, MAR

**Keywords:** ige multiple myeloma, immunoglobulin e, multiple myeloma, plasma cell dyscrasia, rare, t(11.14)

## Abstract

IgE multiple myeloma is an exceptionally unique subtype of plasma cell dyscrasia, accounting for only a marginal percentage of all multiple myeloma cases. Combined with its lack of generalized accepted treatment approaches, its rarity makes diagnosis and clinical management extremely difficult. Here, we report the case of a 50-year-old woman with no prior medical history who presented with progressive bone pain over a one-year period. She underwent an exhaustive diagnostic workup, which included imaging, laboratory workup, histopathology, and cytogenetic analysis. The integrated diagnosis was IgE multiple myeloma associated with t(11;14) translocation. The patient had primary refractoriness to two lines of therapy incorporating immunomodulatory imide drugs and proteasome inhibitors (VRD and CTD regimens). After that, she was started on the DKD protocol consisting of daratumumab (Darzalex) and carfilzomib, with which she experienced a positive clinical and hematologic response. She is now in active surveillance and continues to respond well. Research on IgE multiple myeloma is sparse due to its rarity, but so is research around prognosis, ideal treatment options, and response to newer agents. There is little data because the literature is often based on case reports.

## Introduction

IgE multiple myeloma represents an exceptionally rare subtype, accounting for fewer than 0.01% of all reported cases of multiple myeloma [1]. Since its initial description in 1967, fewer than 70 cases have been documented in the literature up to 2023 [1,2]. It tends to affect relatively younger individuals and shows a clear male predominance [3]. To date, no specific risk factors have been identified or confirmed in the existing literature. The characteristics of patients with IgE multiple myeloma have not been formally compared to those of patients with other multiple myeloma isotypes [4].

The intervention of a laboratory specialist is crucial for diagnosis, as they perform the detection, quantification, and identification of MC in serum and urine [4,5]. They also provide essential data regarding the disease profile, progression, and response to treatment [2]. This helps enable accurate diagnosis, staging, and prognosis, and allows the therapeutic decision-making [5].

Regarding therapeutic strategies, data on efficacy and treatment‑response assessment, particularly with novel agents such as anti‑CD38 monoclonal antibodies and anti‑Bcl‑2 therapies, remain limited [5]. This is primarily due to the scarcity of reported cases, with most available literature consisting of isolated case reports.

## Case presentation

A 52-year-old female patient had a history of a right ankle fracture in 2000 and a left ankle fracture in 2002 following minimal trauma. She had been experiencing low back pain for the past year, associated with inflammatory upper back pain. These symptoms had developed in the context of weight loss quantified at 17 kg and the absence of fever. She visited a rheumatologist in May 2023 for a comprehensive check-up, including routine laboratory tests. Clinical examination revealed a loss of cervical lordosis, an exaggerated thoracic kyphosis, and painful stiffness of the lumbar spine.

Serum protein electrophoresis was requested and revealed a pattern consistent with a moderate inflammatory syndrome and a moderate monoclonal increase in gamma globulins with evidence of distortion. Serum protein immunofixation identified the presence of a monoclonal IgE of the kappa type, along with a monoclonal band detected with anti-kappa light chain antibodies, corresponding to free monoclonal kappa light chains.

Following these findings, the patient was directed to the clinical hematology unit for additional evaluation and management. No evidence of malignancy was identified in the patient’s anamnesis or during the physical examination. A peripheral blood analysis showed normochromic normocytic anemia. There was no vitamin deficiency in the requested assessment, including vitamin B9 and B12 and ferritin. The requested coagulation assessment was normal. Biochemical analysis did not reveal impairment of the liver and renal function or hypercalcemia. Serum laboratory results demonstrated an elevated sedimentation rate and an elevated β2-microglobulin. The lactate dehydrogenase level was normal. Moreover, uric acid and albumin levels were within the normal limits. The urine test revealed a high proteinuria of 2 g on a preserved diuretic. Protein electrophoresis showed the presence of narrow gamma bands.

**Figure 1:**
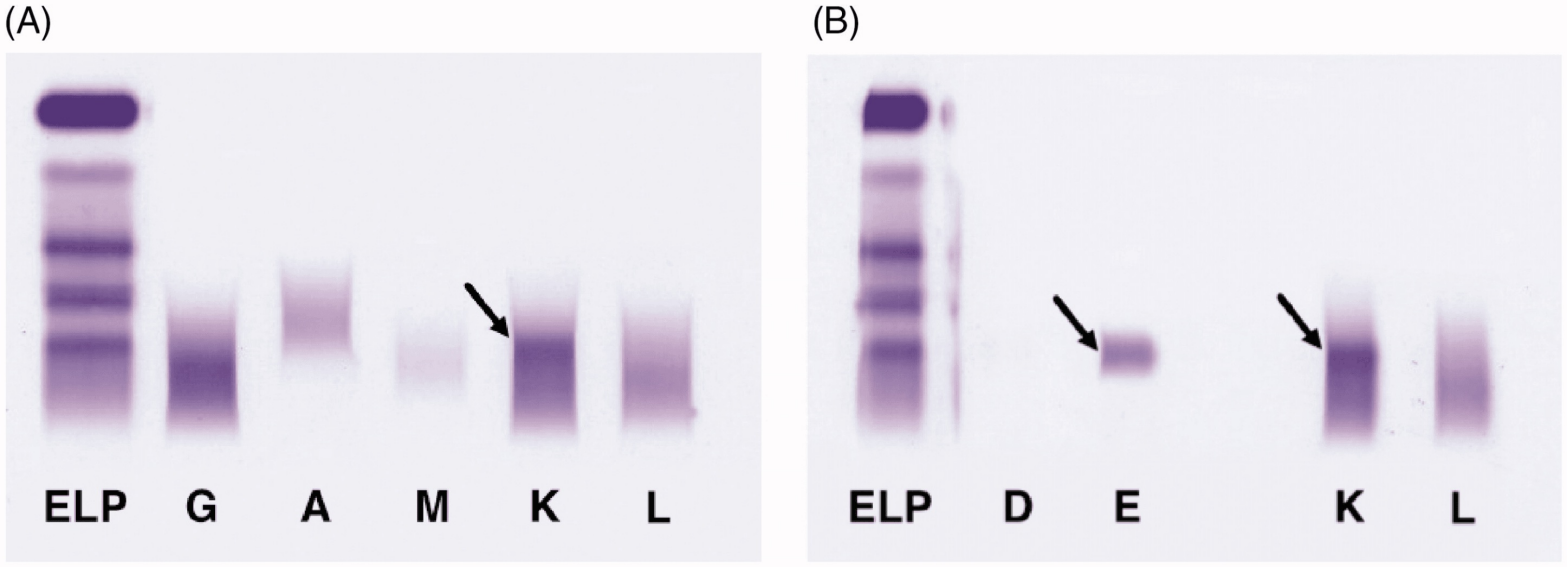
Detection of an IgE kappa paraprotein confirmed through serum protein electrophoresis and immunofixation. (A, B) Serum immunofixation demonstrating the IgE-kappa MC (bands corresponding to the regions of anti-igE and anti-CLL kappa antisera).

The presence of an IgE kappa monoclonal protein was confirmed through serum protein electrophoresis and immunofixation (Figure 1). Serum free light chain assay revealed markedly elevated kappa light chains at 16,279.50 mg/L and lambda light chains at 5.98 mg/L, resulting in a highly abnormal kappa-to-lambda ratio of 790.

Laboratory findings at the time of admission, together with reference values, are summarized in Table 1.

**Table 1:** Laboratory values at the time of admission. MCV: mean corpuscular volume; MCH: mean corpuscular hemoglobin; MCHC: mean corpuscular hemoglobin concentration; Fib: fbrinogen; ALT: alanine aminotransferase; AST: aspartate aminotransferase; CRP: C-reactive protein; Ig: immunoglobulin; LDH: lactate dehydrogenase; PT: prothrombin time; INR: international normalized ratio

	Results	Values of reference
Hemogram		
Erythrocytes, 10^6^/μL	4/10^6^/μL	4.0–5.2
Hemoglobin, g/L	10.7	11.5–15.5
Hematocrit %	32	37–47
MCV, fL	79.1	79–99
MCH, pg	26.6	27–32
MCHC, g/dL	33.6	32–36
Platelets, 10^3^/μL	151	150–400
Leukocytes, ×10^3^/μL	4.8	4.0–10.0
Neutrophils, ×10^3^/μL	2.5	1.5–7
Lymphocytes, 10^3^/μL	1.9	1.0–4.0
Monocytes 10^3^/μL	0.3	0.2–1
Eosinophils, ×10^3^/μL	0.1	0.1–0.4
Basophils, 10^3^/μL	0.0	0–0.1
Biochemistry		
VS mm; 1 hour	90	<20
Creatinine, mg/dL	8.5	5.7–11.1
Urea, mg/dL	0.4	0.15–0.55
Total protein, g/dL	73	64–83
Calcium, mg/dL	90	84–102
β2 microglobulin, mg/L	6	0.97–2.64
Total bilirubin, mg/dL	4	2–12
LDH, U/L	141	125–220
Iron mg/L	1.51	0.5–1.7
Ferritin, ng/mL	245	5–204
Total protein, g/l	76	64–83
Na, mEq/L	140	163–145
K, mEq/L	4.2	3.5–5.1
Cl, mEq/L	106	98–107
AST, U/L	17	5–34
ALT, U/L	21	0–55
Glucose, g/dL	0.84	0.7–1.05
CRP, mg/dL	1.1	<5
Uric acid, mg/L	51.46	26–60
Vitamin B12 pg/mL	200	187–264
Vitamin B9 ng/mL	6.2	3.1–20.5
Troponin, ng/L	<1.3	23–29
24-hour diuresis, L	1.3 l	-
Proteinuria, g /24 hour	2.43 g /24h	0.3
Coagulation		
PT, %	89	70–100
PT (INR)	27	-
Fib, g/dL	5.26	2.5–4
Proteinogram		
Albumin, g/dL	37.52	35–52
α1, g/dL	4.3	2.1–3.5
α2, g/dL	10.3	5.1–8.5
β, g/dL	4.2	3.4–5.2
γ, g/dL	18	8–13.5
CM, g/dL	13.2	-
I light chains in serum		
CLL κ, mg/L	16,279.50	3.30–19.40
CLL λ, mg/L	5.98	5.71–26.30
K/λ ratio	790	0.26–1.65

**Figure 2:**
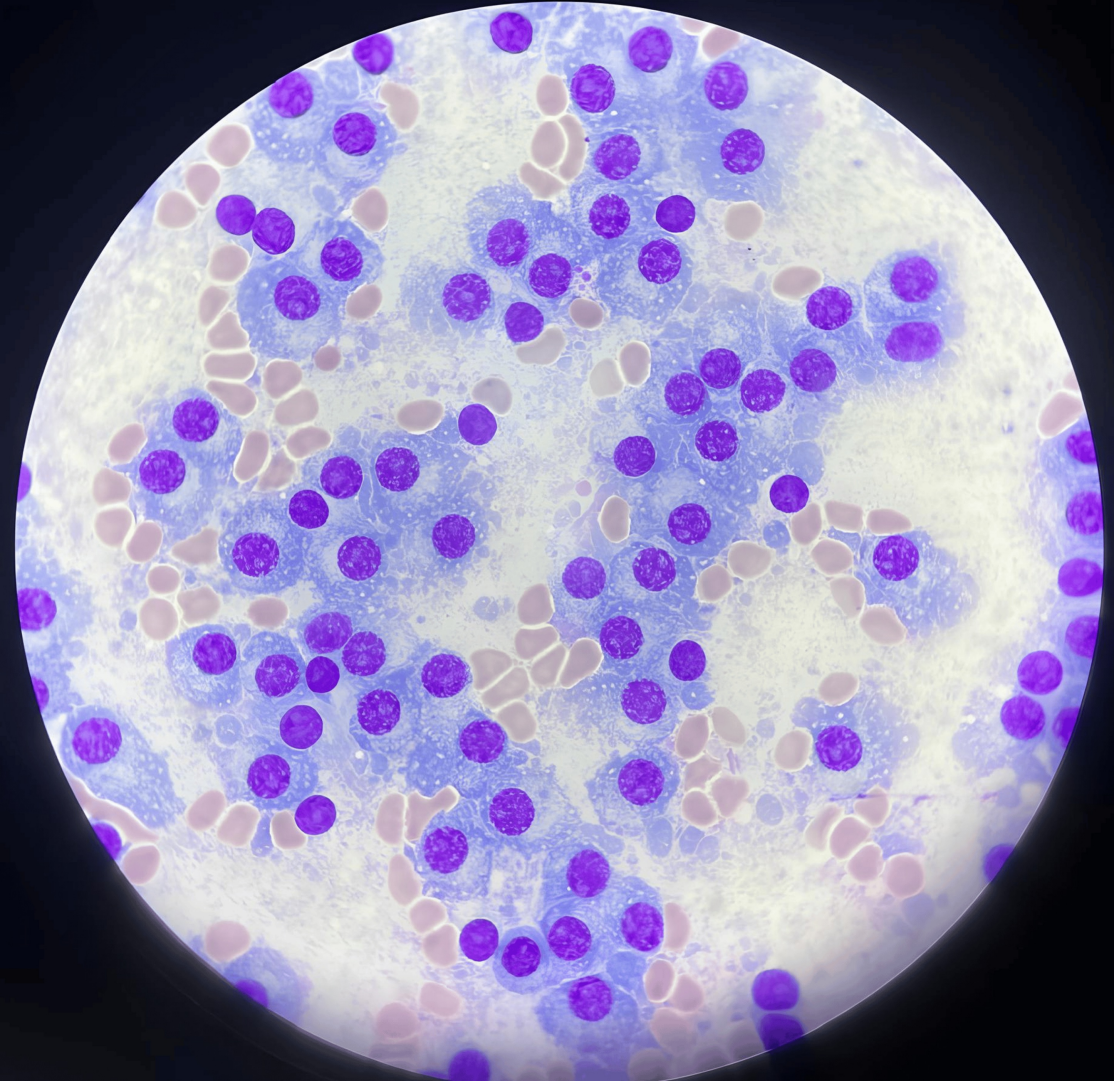
Bone marrow showing a massive infiltration of 75% by dystrophic plasma cells.

Based on the findings, a bone marrow examination was subsequently performed, which showed a massive infiltration of 75% by dystrophic plasma cells (Figure 2).

**Figure 3:**
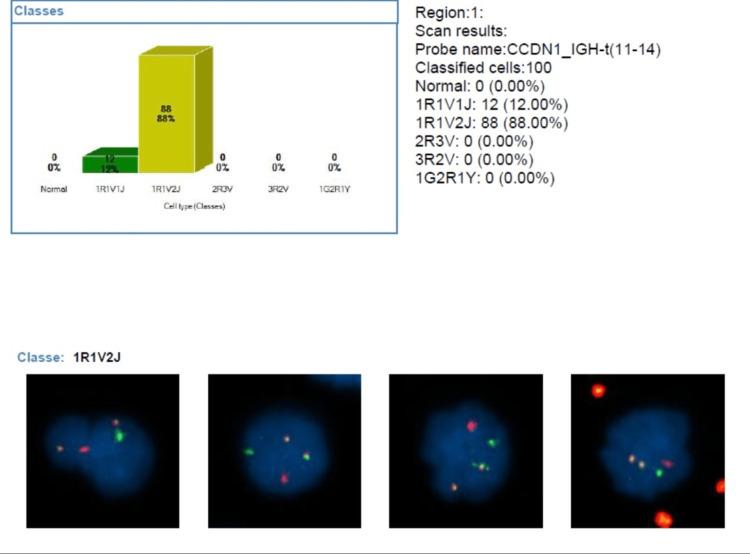
FISH showing the IGH-CCND1 translocation, t(11;14)(q13;q32). Fluorescence in situ hybridization (FISH) with dual fusion probes for immunoglobulin heavy chain (IGH) and cyclin D1 (CCND1) showing a fusion signal corresponding to t(11;14)/IGH-CCND1 fusion, and interphase FISH with a probe for cyclin-dependent kinases regulatory subunit 1 (CKS1B), a chromosome 1q marker, showing four copies of CKS1B.

No cytogenetic aberrations were identified on fluorescence in situ hybridization (FISH) performed on purified plasma cells, with the only abnormality detected being the *IGH-CCND1* translocation, t(11;14)(q13;q32) (Figure 3).

A bone assessment was performed, and radiographic imaging of the thoracolumbar spine revealed multilevel vertebral compression involving both the thoracic and lumbar regions. Thoracolumbar and pelvic MRI confirmed these compressions, which exhibited an osteoporotic pattern, with no imaging features suggestive of multiple myeloma.

Following completion of the diagnostic workup and according to International Myeloma Working Group (IMWG) guidelines, the diagnosis of IgE multiple myeloma was confirmed, stage III according to the International Staging System (ISS) harboring t(11;14), with symptomatic disease based on free light chains, fulfilling the Slim-CRAB diagnostic criteria.

The patient was initially treated according to the VRD protocol, which includes a proteasome inhibitor combined with an immunomodulatory agent and dexamethasone, along with prophylactic treatment: antithrombotic prophylaxis with rivaroxaban, antiviral prophylaxis with valaciclovir, and antibacterial prophylaxis with sulfamethoxazole and trimethoprim.

After four cycles, re-evaluation showed a very good partial response, with serum free light chains showing kappa at 24,462 mg/L, lambda at 11 mg/L, and a kappa/lambda ratio of 2,219.14 mg/L. The patient was scheduled for autologous stem cell transplantation; however, disease progression occurred just before the transplant, with kappa light chains increasing to 4,732 mg/L and the kappa/lambda ratio rising to 591.

Following this progression, the patient was started on second-line therapy using the CTD protocol, which includes cyclophosphamide, thalidomide, and dexamethasone. After six cycles of the CTD protocol, the patient exhibited therapeutic failure, with stable serum free light chain levels, indicating primary refractoriness. Consequently, treatment was switched to the DKD protocol, consisting of daratumumab (Darzalex), carfilzomib, and dexamethasone. The patient showed a favorable response after two cycles, with a decrease in kappa light chains to 50.86 mg/L, lambda light chains to 1.57 mg/L, and a kappa/lambda ratio of 35.72. The patient is currently continuing treatment with the DKD protocol and is showing good clinical progress.

## Discussion

IgE myeloma is an extremely rare subtype of multiple myeloma, accounting for fewer than 0.01% of all multiple myeloma cases. First described in 1967, fewer than 70 cases had been reported in the literature up to 2023. It tends to affect relatively younger patients, with a marked male predominance [6].

The clinical manifestations of IgE myeloma are generally non-specific and, in comparison to other multiple myeloma isotypes, including those with extramedullary involvement, there are no major distinguishing features. However, a notable observation is that most patients are diagnosed at the stage of overt multiple myeloma. Throughout the literature, only two cases of monoclonal gammopathy of undetermined significance involving IgE have been reported. This is likely due to the characteristic small monoclonal spike, often undetectable on standard serum protein electrophoresis, which may contribute to delayed diagnosis [5].

The following features distinguish IgE myeloma [7]: (1) a relatively high frequency of the leukemic form, either at presentation (de novo) or emerging during the disease course; (2) a tendency toward more severe anemia; (3) less frequent occurrences of hypercalcemia and renal impairment; and (4) predominantly osteosclerotic bone lesions (increased bone density/osteocondensation) rather than osteolytic.

IgE myeloma is characterized by small or barely detectable monoclonal IgE spikes on serum protein electrophoresis. Quantitative immunoglobulin assays typically reveal markedly reduced levels of the three major immunoglobulin classes (IgG, IgA, and IgM) [5]. Proteinuria is generally non-massive, not exceeding 0.2 g/L, and approximately 40% of cases involve the presence of free monoclonal kappa light chains. Amyloidosis appears to be rare, according to the literature [1].

Bone marrow examination commonly reveals massive infiltration by dystrophic plasma cells, which can represent up to 80% of marrow cellularity [1]. Cytogenetically, IgE myeloma may exhibit a complex karyotype, frequently showing hyperploidy as well as numerical and structural chromosomal abnormalities. For unknown reasons, t(11;14) is the hallmark of IgE [8].

The possibility of a prozone effect must be taken into account when measuring serum IgE levels [9]. This phenomenon arises from the assay technique, which relies on antigen-antibody interactions balanced to enable detection. In cases where IgE concentrations in the sample are excessively high, saturation of the anti-IgE antibodies may occur, leading to incomplete binding of IgE molecules and impaired formation of detectable immune complexes. Consequently, this can cause underestimation of IgE levels and yield inaccurate results. To avoid such errors, it is essential to perform comprehensive validation across all assays used [4].

The prognosis is generally poor, with a median overall survival of approximately 33 months following conventional treatment, including autologous stem cell transplantation, as reported in a case series of 13 patients with IgE myeloma. This contrasts with a median survival of 62 months typically observed in more common types of multiple myeloma [5].

Regarding treatment efficacy and response assessment, including newer agents such as anti-CD38 monoclonal antibodies and venetoclax, available data remain limited, as the literature is predominantly composed of case reports [10]. A treatment series demonstrated convincingly that combination approaches with novel agents followed by autologous stem cell transplantation for eligible patients constitute an appropriate therapeutic strategy [3].

Some cases of IgE plasma cell neoplasms have responded favorably to novel therapeutic agents, including immunomodulatory imide drugs and proteasome inhibitors. These treatment approaches hold promise for enhancing the prognosis of patients affected by this condition [10]. There is a critical need for novel therapeutic approaches to improve the prognosis of patients with this highly aggressive form of IgE multiple myeloma [11].

The chromosomal translocation t(11;14) represents a key genetic alteration in multiple myeloma that significantly influences therapeutic strategies. This translocation is linked to elevated BCL2 expression, and evidence suggests that t(11;14) predicts a dependency on BCL2 for cell survival [4]. Venetoclax, an oral inhibitor targeting BCL2, has shown encouraging clinical activity in patients with plasma cell neoplasms harboring t(11;14), characterized by high levels of BCL2 expression [12].

Although multiple clinical trials evaluating venetoclax-containing combination regimens in t(11;14)-positive multiple myeloma have been completed or are ongoing, venetoclax remains unapproved for multiple myeloma treatment in any country and is not yet incorporated into routine clinical practice guidelines such as those from the National Comprehensive Cancer Network [13]. Despite increasing reports, there is no consensus on the optimal treatment strategy for IgE multiple myeloma with t(11;14), underscoring the need for multicentric data pooling.

## Conclusions

IgE multiple myeloma is an extremely rare and aggressive form of the disease. Diagnosis can be difficult, and patients often face a tough prognosis. This case highlights the necessity for tailored diagnostic and therapeutic approaches in IgE multiple myeloma, given its distinct biological profile and frequently aggressive course. Despite increasing reports, there is no consensus on the optimal treatment strategy for IgE MM with t(11;14), underscoring the need for further multicenter investigations.
